# Zygotic G2/M Cell Cycle Arrest Induced by ATM/Chk1 Activation and DNA Repair in Mouse Embryos Fertilized with Hydrogen Peroxide-Treated Epididymal Mouse Sperm

**DOI:** 10.1371/journal.pone.0073987

**Published:** 2013-09-10

**Authors:** Bin Wang, Zhiling Li, Chao Wang, Man Chen, Jianfeng Xiao, Xiaoyan Wu, Wanfen Xiao, Yu Song, Xiaoyan Wang

**Affiliations:** 1 Reproductive Center, The First Affiliated Hospital of Shantou University Medical College, Shantou University, Shantou, Guangdong, People’s Republic of China; 2 Reproductive Center, The Affiliated Hospital of Jining Medical College, Jining, Shandong, People’s Republic of China; University of Chicago, United States of America

## Abstract

Human sperm cryopreservation for assisted reproduction is compromised by ROS-induced sperm cryodamage. Our previous model study in which mouse sperm were treated with H_2_O_2_ to simulate sperm DNA-damage caused by cryopreservation-induced ROS have discovered that mouse embryos fertilized with treated sperm showed a delay in cleavage that might be associated with cell cycle arrest. The DNA-damage checkpoint pathway underlying the delay remained elusive. Moreover, our previous study have also indicated that γH2AX, the DNA-damage repair marker, was functional in mouse embryos similarly fertilized, but the completeness and correctness are unknown and warrant more studies because insufficiency of completeness and correctness of DNA repair would otherwise trigger apoptosis. Based on the aforementioned model, we used embryo culture, inverted microscope, BrdU incorporation and immunofluorescence to explore the cell cycle phase that arrest occurred and the underlying DNA-damage checkpoint pathway in mouse zygotes fertilized with H_2_O_2_-treated sperm. We also adopted Tunel to investigate the apoptosis of mouse embryos similarly fertilized at different developmental stages to testify the completeness and correctness of sperm-derived DNA-damage repair. We found G2/M cell cycle arrest in zygotes fertilized with H_2_O_2_-treated sperm. ATM (pSer-1981) and Chk1 (pSer-345) activations, rather than ATR (pSer-428) and Chk2 (pThr-68), were detected in zygotes of the treated group. The apoptosis of embryos of different developmental stages of the treated group weren’t different from those of the untreated group. In conclusions, ATM (pSer-1981)-Chk1 (pSer-345) cascade might have mediated G2/M cell cycle arrest and allowed time to facilitate sperm-derived DNA-damage repair in mouse zygotes fertilized with oxygen-stressed sperm, and the DNA-damage repair might be effective.

## Introduction

Human sperm cryopreservation is a clinical insurance policy against male infertility. However, during cryopreservation sperm are subjected to physical and chemical stresses that can impair their fertilization ability [1]–[6]. Cold shock by sperm cryopreservation is associated with oxidative stress or reactive oxygen species (ROS) generation [7]. Sperm are especially vulnerable to ROS-induced damage, partly because their polyunsaturated fatty acids contents that are favored targets of ROS assaults [8], partly because their deficiencies in antioxidant enzymes resulted from the scarce sperm cytoplasm [8]. Cryopreservation-induced oxidative stress or ROS leads to sperm DNA damage [9]–[11].

Mature sperm are incapable of repairing ROS-induced DNA damage due to loss of repair capabilities during spermatogenesis [12], [13]. Frustratingly, sperm with DNA damage retain the fertilization potential [9]. The DNA damage repair must occur in the embryo in the presences of oocyte-derived transcripts and proteins for correct conveyance of genetic materials to the offspring [9], [12], [14]. Previous studies investigating sperm DNA damage caused by cryopreservation-induced ROS were confined to DNA damage [9], [10]. Little is known about the DNA damage checkpoint pathway whereby embryos fertilized with ROS-stressed sperm react to sperm-derived DNA damage.

Cellular responses to DNA damage involve multiple repair mechanisms and checkpoint responses that delay cell cycle progression, modulate DNA replication and induce apoptosis [15]. Traditionally, the checkpoint pathway is orchestrated primarily through two distinct kinase signaling cascades, ATM-Chk2 and ATR-Chk1 cascades, which are crucial for the proper coordination of checkpoint activation, DNA repairing process and apoptosis [15]. Checkpoint activation transiently halts cell cycle progression and allows time to either repair DNA damage or initiate apoptosis as a last resort if the damage overwhelms the repair mechanisms or was incorrectly or partially repaired [15]–[18]. In eukaryotic cell three checkpoints exist: G1/S, intra-S and G2/M checkpoints [16], [19], [20].

Based on our preestablished DNA-damaged mouse sperm model in which mouse sperm were treated with 1 mM H_2_O_2_ to optimally mimic sperm DNA damage caused by cryopreservation-induced ROS, we have discovered that mouse embryos fertilized with hydrogen peroxide-treated sperm showed a delay in cleavage before the blastocyst stage and γH2AX (The DNA damage repair marker) was functional in the early embryos [21]. The delay prompted us the possibility of cell cycle arrest upon checkpoint activation in early mouse embryos similarly fertilized. We speculate that the cell cycle arrest might most probably occur at the zygotic stage to timely repair sperm-derived DNA damage because embryos would otherwise undergo apoptosis if the damage were left unrepaired [15]–[18]. To deepen our previous study and elucidate the DNA damage checkpoint pathway whereby zygote fertilized with ROS-stressed sperm reacts to sperm-derived DNA damage, we herein used the preestablished DNA-damaged mouse sperm model to further explore the cell cycle arrest in mouse zygotes fertilized with ROS-stressed sperm, and the possible DNA damage checkpoint pathway underlying the cell cycle arrest was also investigated.

Despite our previous parallel study demonstrated that γH2AX (The DNA damage repair marker) was functional in mouse embryos fertilized with hydrogen peroxide-treated sperm [21], the completeness and correctness of it remain unknown. As stated above, after DNA damage sensing, cell cycle arrest mediated by checkpoint activation would proceed to apoptosis if the damage overwhelmed the repair mechanisms or was incorrectly or partially repaired [15]–[18]. To testify this, we therefore surveyed the apoptosis of mouse embryos similarly fertilized at different developmental stages.

## Materials and Methods

### Mice

Adult Kun-Ming mice (3–6 weeks old) were purchased from the animal center of Shantou University Medical College and treated in compliance with The Guide for the Care of Use of Laboratory Animal by the US National Institutes of Health (NIH Publication No. 85-23, revised 1996) and the rules of the National Animal Protection of China. All experimental protocols were approved by the Laboratory Animal Ethics Committee of our institution (SUMC2011-107). This study was approved by the Institutional Animal Care and Use Committee of Shantou University Medical College.

### Epididymal Sperm Preparation, Collection and Culture of Oocytes and Embryos

As described in our previous study [21], sperm were collected from the caudae epididymis of mice and incubated in capacitation medium (HTF medium [CooperSurgical, Inc.] containing 1.5% BSA) at 37°C in a 5% CO_2_ incubator for 1 h. Female mice were superovulated with consecutive injection of 10 IU pregnant mare serum gonadotropin and 10 IU human chorionic gonadotropin 48 h apart, sacrificed at 13 to 15 h after the human chorionic gonadotropin administration. Fully grown germinal vesicle oocytes were obtained from the ovaries of the mice. Cumulus oocytes were collected in 37°C PBS, then moved to 37°C fertilization liquid (HTF medium containing 0.4% BSA) and washed further. The oocytes were then inseminated with sperm as prepared above in HTF medium under the conditions of 37°C and 5% CO_2_. 4 hours post insemination; the embryos were washed with 37°C HTF medium, and then incubated in embryo culture medium (HTF medium containing 0.4% BSA and 10% fetal bovine serum) under the same conditions with medium renewed daily. For blastocyst culture, the embryo culture medium was replaced with blastocyst medium (CooperSurgical, Inc.).

### DNA-damaged Mouse Sperm Model Induced by Hydrogen Peroxide

As described in our previous study [21], treatment of sperm with capacitation medium containing 1 mM H_2_O_2_ induced DNA damage that resembled sperm DNA damage caused by cryopreservation-induced ROS. In this study, we continued to use this preestablished DNA-damaged mouse sperm model to simulate sperm DNA damage resulted from cryopreservation-induced ROS. Embryos fertilized with H_2_O_2_-stressed sperm were defined as the treated group, and those fertilized with fresh sperm were defined as the untreated control group.

### Determination of the Onset of G1 Phase, the Onset and Endpoint of S Phase and the Endpoint of M Phase in Mouse Zygotes

According to previous studies [22], [23], we used the time point when the second polar body was emitted from the zygote as the onset of G1 phase. Observation of the onset of G1 phase was conducted under the inverted microscope (Olympus Inc., Japan) from 1 h to 5 h post insemination, with observation performed every other 30 min. 78 zygotes of the treated group and 61 zygotes of the untreated control group were observed for the onset of G1 phase. The onset and endpoint of S phase was determined with BrdU incorporation method as described below. Determination was done from 8 h to 19 h post insemination, with 15∼25 zygotes collected and assessed every other 1 h. The onset of S phase was determined when more than 10% of the total zygotes assessed were BrdU-positive, and the endpoint of it was determined when more than 90% of the previous BrdU-positive zygotes lost positivity. 233 zygotes of the treated group and 163 zygotes of the untreated control group were respectively determined for the onset and endpoint of S phase. The endpoint of M phase was assessed as the time point when zygotic embryo cleaved. 222 zygotes of the treated group and 185 zygotes of the untreated control group were respectively determined for the endpoint of M phase.

### BrdU Incorporation Test

The mouse zygotes were cultivated in embryo culture medium at 37°C in a 5% CO_2_ incubator. Starting from 8 h to 19 h post insemination, 15∼25 zygotes of the treated and the untreated control groups were respectively retrieved every other 60 min, then incubated under the same conditions for 30 min in embryo culture medium supplemented with 1 mM BrdU as optimized by our preliminary experiments. The zygotes were fixed in 2.5% paraformaldehyde for 15 min and mounted on polylysine slides, washed with washing solution (PBS containing 10% FBS and 0.2% tritonX-100), incubated for 30 min in 1 mM HCl, washed for 20 min in 0.1 M borate buffer solution, washed again with washing solution, blocked in washing solution for 30 min at 37°C, then incubated with 6 µg/ml anti-BrdU antibody (Sigma) for 1 h at 37°C. Subsequently the slides were washed with PBS containing 2% FBS and 0.1% tritonX-100, incubated with FITC-conjugated goat anti-mouse IgG antibody (Invitrogen) for 1 h at 37°C, washed with washing solution, counterstained with 10 µg/ml propidium iodide (Beyotime) overnight at 4°C, washed again with washing solution and sealed with coverslips and mounting medium (Beyotime). Signal observation was done with an Olympus FluoView FV 1000 confocal microscope (Olympus Inc., Japan). The number of BrdU-positive zygotes and total zygotes assessed were counted. The BrdU-positive rate was defined as BrdU-positive rate = the number of BrdU-positive zygotes/the total number of zygotes assessed.

### Immunofluorescence

To explore the DNA damage checkpoint pathway underlying the possible cell cycle arrest in mouse zygotes fertilized with hydrogen peroxide-treated sperm, we investigated the activations of relevant regulatory proteins, i.e. ATM (pSer-1981), ATR (pSer-428), Chk1 (pSer-345) and Chk2 (pThr-68). The zygotes were collected and washed with TPBS (PBS supplemented with 0.05% tween-20), then digested with 0.1% pancreatin to remove zonae pellucidae. After washing with TPBS, the zygotes were fixed in 4% paraformaldehyde for 30 min and mounted on polylysine slides, washed again with TPBS. The zygotes were permeablized with TPBS supplemented with 0.5% TritionX-100 at room temperature for 30 min, washed with TPBS and blocked for 1 h at room temperature in blocking solution (For ATM and ATR, blocking solution was TPBS containing 3% BSA and 10% goat serum, For Chk1 and Chk2, it was TPBS containing 3% BSA and 10% donkey serum). The primary antibodies (Anti-ATM protein kinase pSer-1981 monoclonal antibody, Rockland; p-ATR (Ser-428), Santa Cruz Biotechnology; p-Chk1 (Ser-345), Santa Cruz Biotechnology; anti-Chk2 (phospho T68) antibody, Abcam) were then applied and incubated overnight at 4°C, washed with TPBS and incubated with secondary antibodies at room temperature for 1 h (For ATM: Alexa Fluor 488 goat anti-mouse IgG (H+L), Invitrogen; for ATR: goat anti-rabbit IgG-FITC, Santa Cruz Biotechnology; for Chk1: donkey anti-goat IgG-FITC, Santa Cruz Biotechnology; for Chk2: donkey anti-rabbit IgG-TR, Santa Cruz Biotechnology). For ATM and Chk1, the zygotes were washed with TPBS and counterstained with propidium iodide at room temperature for 30 min, washed again and sealed with coverslips and mounting medium (Beyotime); For ATR and Chk2, the zygotes were washed with TPBS and sealed with coverslips and mounting medium containing DAPI (Sigma). Signal observation was done with an Olympus FluoView FV 1000 confocal microscope (Olympus Inc., Japan).

### Tunel Assay

To survey the apoptosis of mouse embryos fertilized with hydrogen peroxide-treated sperm at different developmental stages, Tunel assay was performed with the In Situ Cell Death Fluorescein Kit (Roche) in accordance with manufacturer’s instruction. The embryos were withdrawn 17, 24, 48, 60, 72, 84 and 96 h post insemination to optimally collect zygotic embryos, 2-cell embryos, 4-cell embryos, 8-cell embryos, morulae, early blastocysts and blastocysts according to our preliminary experiments. Zonae pellucidae were first removed from the embryos. The embryos were then washed with TPBS, fixed in 4% paraformaldehyde at room temperature for 30 min and mounted on polylysine slides, washed again with TPBS and permeabilized in TPBS containing 0.5% TritonX-100 at room temperature for 30 min. The embryos were washed with TPBS and incubated with fluorescein-conjugated dUTP and terminal deoxynucleotidyl transferase in darkness at 37°C for 1 h. The reaction was terminated by washing in TPBS for 15 min, and then the embryos were counterstained and sealed with coverslips and mounting medium containing DAPI (Sigma). Observation was done under the Olympus FluoView FV 1000 confocal microscope (Olympus Inc., Japan). The apoptotic rate for each embryo was expressed as the percentage of Tunel-positive cell number relative to the total cell number of the embryo as defined in previous studies [24], [25]. The number of treated and untreated embryos at each developmental stage were shown as follows: 226 and 224 for zygotic embryos, 176 and 191 for 2-cell embryos, 163 and 176 for 4-cell embryos, 137 and 156 for 8-cell embryos, 87 and 102 for morulae, 86 and 95 for early blastocysts and 63 and 80 for blastocysts.

### Statistical Analysis

Data are shown as mean±SD. For continuous data, including the onset of G1 phase, the onset of S phase, the endpoint of S phase and the onset of M phase, t-test was used to analyze the differences of these data between the treated and the untreated control groups. For categorical data apoptotic cell number, Chi square test was used to compare the composition difference of apoptotic cell number in the two groups. P<0.05 was considered statistically significant. Data analysis involved use of SPSS 13.0 (SPSS Inc., USA).

## Results

### The Onset of G1 Phase, the Onset and Endpoint of S Phase and the Endpoint of M Phase of Mouse Zygotes in the Treated and the Untreated Control Groups

As revealed in Table 1, the onsets of G1 phase of the treated and the control groups were 1.7±1.1 hpi (Hours post insemination) and 1.8±1.0 hpi, showing no significant difference (p>0.05). The onsets of S phase were 10.3±1.0 hpi and 9.8±0.6 hpi for the treated and the control group respectively, no significant difference was found (p>0.05). Likewise, the endpoints of S phase of the treated and the control groups were 17.7±0.6 hpi and 17.0±0.8 hpi, no significant difference was present (p>0.05). However, the endpoints of M phase of the treated and the control groups were 22.5±1.1 hpi and 20±0.9 hpi, displaying significant difference (p<0.05).

**Table 1:** The onset of G1 phase, the onset and endpoint of S phase and the endpoint of M phase of mouse zygotes in the untreated control and the treated groups.

Characteristics	The controlgroup	The treatedgroup	p value
The onset of G1 phase	1.80±1.0 hpi	1.70±1.1 hpi	>0.05
The onset of S phase	9.80±0.6 hpi	10.3±1.0 hpi	>0.05
The endpoint of S phase	17.0±0.8 hpi	17.7±0.6 hpi	>0.05
The endpoint of M phase	20.0±0.9 hpi	22.5±1.1 hpi*	<0.05

Data are expressed as mean±SD, hpi: hours post insemination. Zygotes fertilized with hydrogen peroxide-stressed sperm were defined as the treated group, and those fertilized with fresh sperm were defined as the untreated control group.

* p<0.05: the treated group vs. the untreated control group, t-test was used.

### Activations of ATM (pSer-1981), ATR (pSer-428), Chk1 (pSer-345) and Chk2 (pThr-68) of Mouse Zygotes in the Treated and the Untreated Control Groups

**Figure 1:**
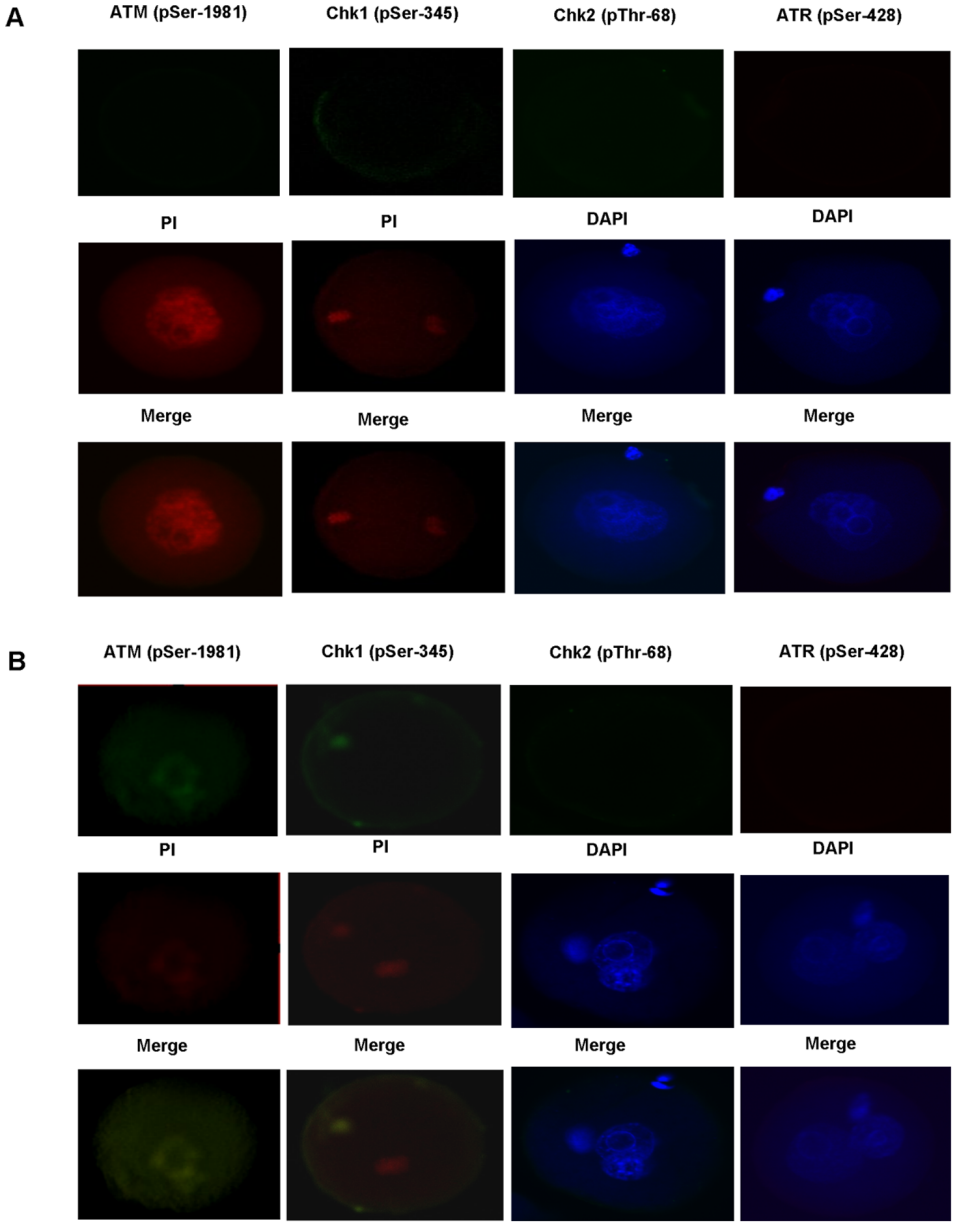
Activations of ATM (pSer-1981), ATR (pSer-428), Chk1 (pSer-345) and Chk2 (pThr-68) of mouse zygotes in the untreated control group and the treated group. The treated group: zygotes fertilized with hydrogen peroxide-stressed sperm, the untreated control group: zygotes fertilized with fresh sperm. PI: Propidium iodide, DAPI: 4′, 6-diamidino-2-phenylindole. Positive signals (Green stains) for ATM (pSer-1981) and Chk1 (pSer-345) were respectively observed in the 1^st^ and the 2^nd^ columns of the left side of Panel B, indicatives of ATM (pSer-1981) and Chk1 (pSer-345) activations in mouse zygotes of the treated group. No positive signals (Green stains) were detected in other columns of Panel A and Panel B. Nuclei were stained with PI (Red) or DAPI (Blue).

We detected ATM (pSer-1981) and Chk1 (pSer-345) activations in mouse zygotes of the treated group (Figure 1B). However, we did not detect ATR (pSer-428) and Chk2 (pThr-68) activations in mouse zygotes of the treated group (Figure 1B). In contrast to the treated group, ATM (pSer-1981), ATR (pSer-428), Chk1 (pSer-345) and Chk2 (pThr-68) activations weren’t found in mouse zygotes of the control group (Figure 1A).

### Apoptosis of Mouse Embryos of Different Developmental Stages in the Treated and the Untreated Control Groups

**Figure 2:**
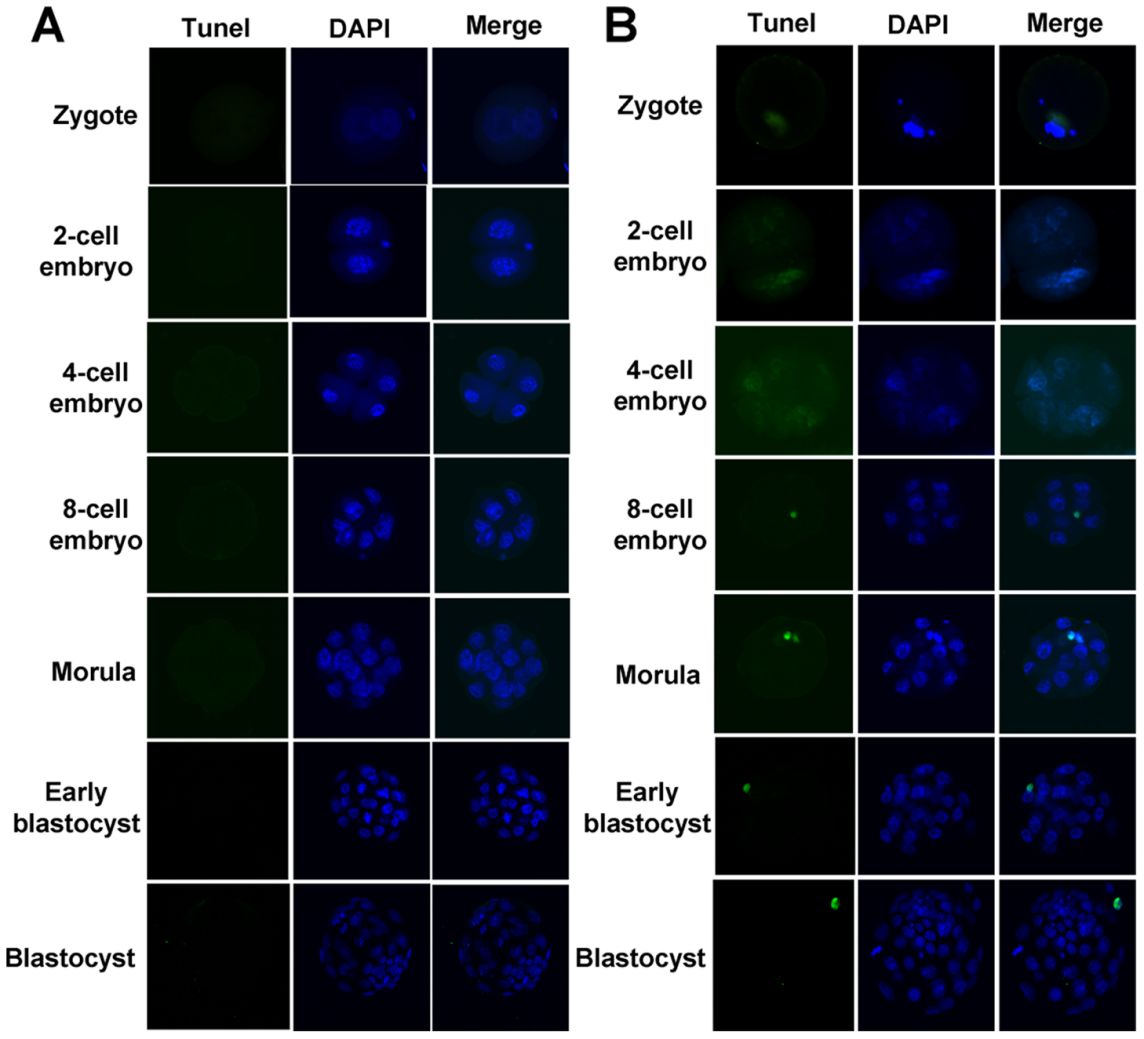
Representative images of normal and apoptotic mouse embryos at different developmental stages. Tunel: Terminal-deoxynucleotidyl transferase mediated nick end labeling. DAPI: 4′, 6-diamidino-2-phenylindole. Panel A are representative images of normal mouse embryos at different developmental stages, no positive signals (Green stains) were detected in each of the seven rows of Panel A. Panel B are representative images of apoptotic mouse embryos at different developmental stages, positive signals (Green stains) were observed in each of the seven rows of Panel B. Nuclei were stained with DAPI (Blue).

As shown in Table 2, the average apoptotic rates of zygotes, 2-cell embryos, 4-cell embryos, 8-cell embryos, morulae, early blastocysts and blastocysts of the treated group were 1.77±0.88, 2.00±0.84, 1.43±0.47, 1.02±0.30, 2.98±0.47, 2.20±0.35 and 1.66±0.36 respectively as compared to 0.00±0.00, 0.96±0.48, 0.87±0.36, 0.56±0.21, 1.84±0.31, 1.59±0.28 and 1.28±0.29 of the control group. For each of the seven developmental stages assessed, the apoptosis of mouse embryos fertilized with hydrogen peroxide-treated sperm was higher than that of the control group, but no significant difference was found as indicated in Table 2. Representative images of normal and apoptotic mouse embryos of different developmental stages are illustrated in Figure 2A and Figure 2B respectively.

**Table 2:** Apoptosis of mouse embryos of different developmental stages in the untreated control and the treated groups.

Embryo stage	Culture period(hpi)	Embryo number assessed		Average cell number per embryo		Average apoptotic cell number per embryo		Average apoptotic rate (%)	
		The control group	The treated group	The control group	The treated group	The control group	The treated group	The control group	The treated group
Zygote	17	224	226	1.00±0.00	1.00±0.00	0.00±0.00	0.02±0.01*	0.00±0.00	1.77±0.88
2-cell embryo	24	191	176	2.05±0.05	1.98±0.04	0.02±0.01	0.03±0.01*	0.96±0.48	2.00±0.84
4-cell embryo	48	176	163	3.88±0.05	3.76±0.06	0.03±0.01	0.06±0.02*	0.87±0.36	1.43±0.47
8-cell embryo	60	156	137	7.95±0.03	7.85±0.05	0.04±0.02	0.08±0.02*	0.56±0.21	1.02±0.30
Morula	72	102	87	16.37±0.31	15.97±0.33	0.28±0.04	0.44±0.07*	1.84±0.31	2.98±0.47
Early blastocyst	84	95	86	27.02±0.96	26.05±0.91	0.39±0.07	0.52±0.08*	1.59±0.28	2.20±0.35
Blastocyst	96	80	63	55.78±1.98	51.56±1.78	0.61±0.12	0.83±0.16*	1.28±0.29	1.66±0.36

Data are presented as mean±SD, hpi: hours post insemination. The apoptotic rate for each embryo was expressed as the percentage of apoptotic cell number relative to the total number of the embryo. Embryos fertilized with hydrogen peroxide-stressed sperm were defined as the treated group, and those fertilized with fresh sperm were defined as the untreated control group.

* p>0.05, the treated group vs. the untreated control group, Chi square test was use to compare the composition difference of apoptotic cell number of the two groups.

## Discussion

Human sperm cryopreservation is of great importance in clinical management of male infertility; however it is complicated by cryopreservation-induced sperm cryodamage during the freeze-thaw cycle [26]. Many previous studies exploring cryopreservation-induced sperm cryodamage were restricted to motility, viability, acrosome status, fertilizing ability, sperm apoptosis, sperm DNA damage, etc [2]–[6], [9]–[11]. Although numeric studies have examined sperm DNA damage caused by cryopreservation-induced ROS [9], [10], limited information exists about the DNA damage checkpoint pathway whereby zygotes fertilized with ROS-stressed sperm react with sperm-derived DNA damage. The aim of this study is to shed some lights on this unsolved puzzle.

Based on our preestablished DNA-damaged mouse sperm model [21], this study found that in mouse zygotes fertilized with hydrogen peroxide-treated sperm the onset of G1 phase wasn’t different from that of the untreated control group, so did the onset of S phase and the endpoint of S phase (Table 1, all p>0.05). But significant difference existed in the endpoint of M phase between the treated and the untreated control groups (Table 1, p<0.05). Our data indicated that mouse zygotes fertilized with oxygen-stressed sperm responded to sperm-derived DNA damage via induction of G2/M cell cycle arrest, rather than G1 or intra-S arrest. This finding coincides with our previous study which demonstrated that mouse embryos fertilized with oxygen-stressed sperm showed a delay in cleavage before the blastocyst stage [21].

Furthermore, our study showed that mouse zygotes fertilized with hydrogen peroxide-treated sperm continued to develop into blastocysts after the G2/M cell cycle arrest (data reflected in the third part of the Results section), suggesting that in the zygotes G2/M DNA damage checkpoint mechanism was effective in responding to the sperm-derived DNA damage. [21]. Our finding might seem to contradict with the study by M Yukawa et al which indicated that G2/M DNA damage checkpoint mechanism functioned insufficiently in mouse zygotes irradiated with 10 Gy γ-rays and subsequently resulted in embryonic developmental arrest [27]. However, the discrepancy could be explained by the variation in the extent of DNA damage. The DNA damage induced by 1 mM H_2_O_2_ in our study would seem minor as compared to that by 10 Gy γ-rays in the study by M Yukawa et al [27]. G2/M checkpoint mechanism is limited in mouse zygote because its functioning relies on the limited oocyte-derived transcripts and proteins [27]. Our study suggested that in mouse zygote the limited G2/M checkpoint pathway was able to cope with cryopreservation-induced sperm-derived DNA damage.

Cell cycle arrest is coordinated by a DNA damage checkpoint pathway that comprises two core kinase signaling cascades, ATM-Chk2 and ATR-Chk1 cascades [15]. Our study also explored the possible kinase signaling cascade underlying the zygotic G2/M cell cycle arrest observed and examined the activations of relevant regulatory kinase proteins ATM (pSer-1981), ATR (pSer-428), Chk1 (pSer-345) and Chk2 (pThr-68) in mouse zygotes fertilized with hydrogen peroxide-treated sperm (The treated group) and those fertilized with fresh sperm (The control group). Intriguingly, as revealed in Figure 1B, in the treated group we detected ATM (pSer-1981) and Chk1 (pSer-345) activations, but we did not detected ATR (pSer-428) and Chk2 (pThr-68) activations. In contrast, ATM (pSer-1981), ATR (pSer-428), Chk1 (pSer-345) and Chk2 (pThr-68) activations weren’t detected in the control group (Figure 1A).

Historically, ATM-Chk2 and ATR-Chk1 cascades are thought to act in parallel with Chk1 and Chk2 playing overlapping or partially redundant roles in downstream checkpoint responses [15]. However recent reports indicated a crosstalk between these two cascades [28], [29]. ATM was able to relay signal through Chk1 activation [29]–[31]. G2/M cell cycle arrest by Chk1 (pSer-345) activation was previously reported in human prostate, ovarian and hepatocyte [32]–[35]. More importantly, RP Sahu et al found that H2AX (pSer-139), ATM (pSer-1981) and Chk1 (pSer-345) activations were concomitant with G2/M cell cycle arrest in curcumin-treated human pancreatic cancer cells, but they did not observed ATR (pSer-428) and Chk2 (pThr-68) activations [36]. RP Sahu et al also found that silencing ATM or Chk1 expression abrogated ATM (pSer-1981) and Chk1 (pSer-345) activations in curcumin-treated human pancreatic cancer cells and subsequently prevented the cells from undergoing G2/M cell cycle arrest [36]. Our finding is in agreement with these results by underscoring the critical role of ATM-Chk1 cascade in mediating G2/M cell cycle arrest [36]. Considering the aforementioned facts, we believe that mouse zygotes fertilized with oxygen-stressed sperm might have activated ATM (pSer-1981)-Chk1 (pSer-345) cascade to mediate G2/M cell cycle arrest to gain time for repair of sperm-derived DNA damage. Our study is the first to elucidate the DNA damage checkpoint pathway whereby zygotes fertilized with oxygen-stressed sperm react with sperm-derived DNA damage. However, the DNA damage checkpoint pathway might quite likely be a vast and complex network, further investigations would be necessary to better unveil this unsolved puzzle.

Several studies showed that after DNA damage sensing, cell cycle arrest mediated by checkpoint activation would proceed to apoptosis if the damage overwhelmed the repair mechanisms or was incorrectly or partially repaired [14]–[18]. Therefore, we surveyed the apoptosis of mouse embryos fertilized with hydrogen peroxide-treated sperm at seven different developmental stages to study the completeness and correctness of the DNA damage repair. Our result indicated that for each of the seven developmental stages assessed, the apoptosis of mouse embryos fertilized with hydrogen peroxide-treated sperm was higher than that of the control group, but no significant difference was found (Table 2). The apoptosis data herein act in concert with our previous parallel study to suggest that DNA repair in embryos fertilized with oxygen-stressed sperm might be effective [21].

In conclusion, our study suggested that ATM (pSer-1981)-Chk1 (pSer-345) cascade might have mediated G2/M cell cycle arrest to allowed time to facilitate sperm-derived DNA damage repair in mouse zygotes fertilized with oxygen-stressed sperm. DNA repair in embryos similarly fertilized might be effective.
